# Improving Tensile and Compressive Properties of an Extruded AZ91 Rod by the Combined Use of Torsion Deformation and Aging Treatment

**DOI:** 10.3390/ma10030280

**Published:** 2017-03-10

**Authors:** Bo Song, Chunpeng Wang, Ning Guo, Hucheng Pan, Renlong Xin

**Affiliations:** 1 Faculty of Materials and Energy, Southwest University, Chongqing 400715, China; whc34@swu.edu.cn; 2 College of Materials Science and Engineering, Chongqing University, Chongqing 400044, China; c.p.wang996@gmail.com; 3 Key Laboratory for Anisotropy and Texture of Materials (Ministry of Education), College of Materials Science and Engineering, Northeastern University, Shenyang 110819, China; panhc@atm.neu.edu.cn

**Keywords:** AZ91 alloy, aging treatment, torsion deformation, gradient microstructure, yield asymmetry

## Abstract

In this study, AZ91 magnesium alloy rods were used to investigate the effects of torsion deformation on microstructure and subsequent aging behavior. Extruded AZ91 rod has a uniform microstructure and typical fiber texture. Torsion deformation can generate a gradient microstructure on the cross-section of the rod. After torsion, from the center to the edge in the cross-section of the rod, both stored dislocations and area fraction of {10-12} twins gradually increase, and the basal pole of the texture tends to rotate in the ED direction. Direct aging usually generates coarse discontinuous precipitates and fine continuous precipitates simultaneously. Both twin structures and dislocations via torsion deformation can be effective microstructures for the nucleation of continuous precipitates during subsequent aging. Thus, aging after torsion can promote continuous precipitation and generate gradient precipitation characteristics. Both aging treatment and torsion deformation can reduce yield asymmetry, and torsion deformation enhances the aging hardening effect by promoting continuous precipitation. Therefore, combined use of torsion deformation and aging treatment can effectively enhance the yield strength and almost eliminate the yield asymmetry of the present extruded AZ91 rod. Finally, the relevant mechanisms are discussed.

## 1. Introduction

As the lightest metallic structural materials, Mg alloys are expected to replace some steel and aluminum alloys in vehicles for the purpose of weight reduction. However, the improvement of strength and anisotropy is still needed to extend the applications of Mg alloys. Precipitation hardening is an important hardening method and has exhibited high competitiveness in the development of high strength Mg alloys [1,2]. Moreover, it has been reported that the characteristics of precipitates, such as size, morphology and distribution, strongly influence the precipitation hardening effect and mechanical anisotropy [1,3,4,5,6,7,8]. Therefore, an increasing amount of work has been focused on the controlling of precipitation characteristics.

Cold deformation is an effective way to tailor the precipitation behavior of Mg alloys [8,9,10,11,12,13]. It has been reported that the high density of dislocations generated by cold working accelerated aging kinetics and facilitated the nucleation of precipitates during the aging process, leading to a higher aging hardening response [9]. Moreover, twinning deformation could also influence the precipitation behavior of Mg alloys. For Mg alloys, cold deformation along appropriate directions can easily produce a large number of {10-12} extension twins instead of dislocations [14]. Twin boundaries could be favorable nucleation sites for precipitates [15,16]. Some works also found that pre-twinning deformation promotes continuous precipitation and suppresses the discontinuous precipitation in AZ80 and AZ91 alloys [8,11,17]. In addition, dislocations, textural change and twin lamellae via cold deformation can also be used to tailor strength-toughness properties and anisotropy of Mg alloys [18,19]. Therefore, the combined use of cold deformation and aging treatment has become a new strategy to fabricate Mg alloys with good properties.

In previous studies, pre-cold deformation is usually carried out by pre-tension, pre-compression and pre-rolling [8,9,10,19,20,21]. Recently, it has been found that torsion deformation is somewhat superior for improving the mechanical properties of rod-shaped materials [22,23,24,25,26,27,28]. Specifically, torsion deformation can introduce a gradient distribution of deformed microstructure compared with other deformation types. Thus, it is very interesting to investigate the effect of torsion deformation on precipitation characteristics of Mg alloys. AZ91 alloy is a typical precipitation-hardenable Mg alloy and has garnered much research interest due to its high strength and low cost [3,29]. In this study, an extruded AZ91 rod is used. Microstructure evolution during torsion deformation and its influence on precipitation characteristics during subsequent aging are investigated. It is found that torsion deformation can effectively promote continuous precipitation and fabricate an Mg alloy rod with gradient precipitation characteristics. It is proven that the combined use of torsion deformation and aging treatment can enhance the aging hardening effect and eliminate yield asymmetry in the extruded AZ91 rod. Finally, the relevant mechanisms are discussed.

## 2. Experimental Section

**Figure 1:**
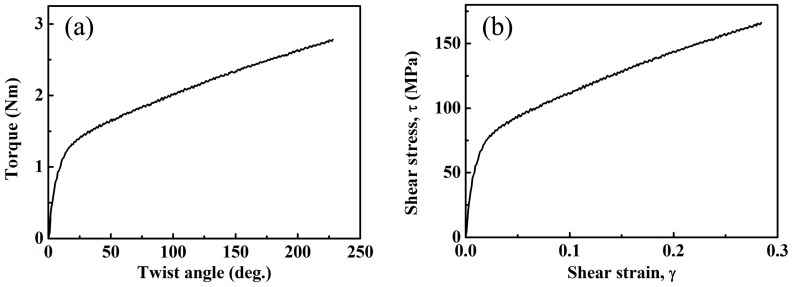
(**a**) Measured twist angle vs. torque curve of AZ91 during free end torsion at room temperature and (**b**) shear stress vs. shear strain curve obtained from the measured twist angle vs. torque curve.

The starting AZ91 material was an extrusion billet. Cylinders with a dimension of 85 mm × 180 mm were cut from the as-cast AZ91 alloy and homogenized at 400 °C for 24 h and then hot-extruded into rods with a diameter of 16 mm. The extrusion ratio was about 28:1 and extrusion rate was about 20 mm·s^−1^. The temperature of the ingots was kept at 400 °C during the whole extrusion process. Extruded AZ91 rods were solution treated at 420 °C for 3 h, then quenched into water at room temperature. The solution-treated AZ91 rods were subjected to free-end torsion deformation. Dog-bone-shaped specimens with a gauge length as Φ 4 mm × 28 mm were prepared for the torsion deformation. The longitudinal axis of the specimen is parallel to the extrusion direction (ED). The torsion test was carried out at a constant rate of 2 rpm, which resulted in a maximum equivalent strain rate of 0.9 × 10^−2^ s^−1^ at the sample surface. Figure 1a displays the torque-twist angle curve. According to previous literature [30], shear stress versus shear strain curves can be calculated from measured torque versus twist angle curves and are shown in Figure 1b. It indicates that the shear stress increases with the shear strain via the work hardening effect and the sample finally fractures when the twist angle increases to approximately 220°. In this study, the extruded rod is designed to twist to an angle of 180° at room temperature. Part of these samples suffered aging treatment at 180 °C for 36 h. In total, four types of samples were prepared in this study. The processing histories of the four types of rods are listed in Table 1.

Tensile and compressive tests along ED of the twisted samples were performed at room temperature at a strain rate of 1 × 10^−3^ s^−1^. After torsion, some of the dog-bone-shaped specimens were used for the tension test, and the cylindrical specimens with nominal dimensions of Φ 4 mm × 8 mm were cut from the dog-bone-shaped specimens for the compression test. The yield strength was measured as 0.2% proof stress and the extent of yield asymmetry was described as the ratio of compressive yield stress to tensile yield stress (CYS/TYS).

Microstructures were characterized using scanning electron microscopy (SEM, JEOL, Tokyo, Japan) and electron backscatter diffraction (EBSD). EBSD measurements were carried out on cross-sections of the samples and characterized regions were near the surface of rods (edge position) and near the cores of rods (center position). The characterized region for edge position is about 1.8 mm from the rod axis and suffers a shear strain of approximately 20%. Surfaces of samples for EBSD measurement were prepared by mechanical grinding using silicon carbide papers, followed by electro-polishing in the AC2 electrolyte for 60 s at 20 V. EBSD analyses were carried out by using the HKL Channel-5 software (AZtecHKL, Oxford Instruments, London, UK).

## 3. Results

### 3.1. Microstructure Evolution During Torsion

**Figure 2:**
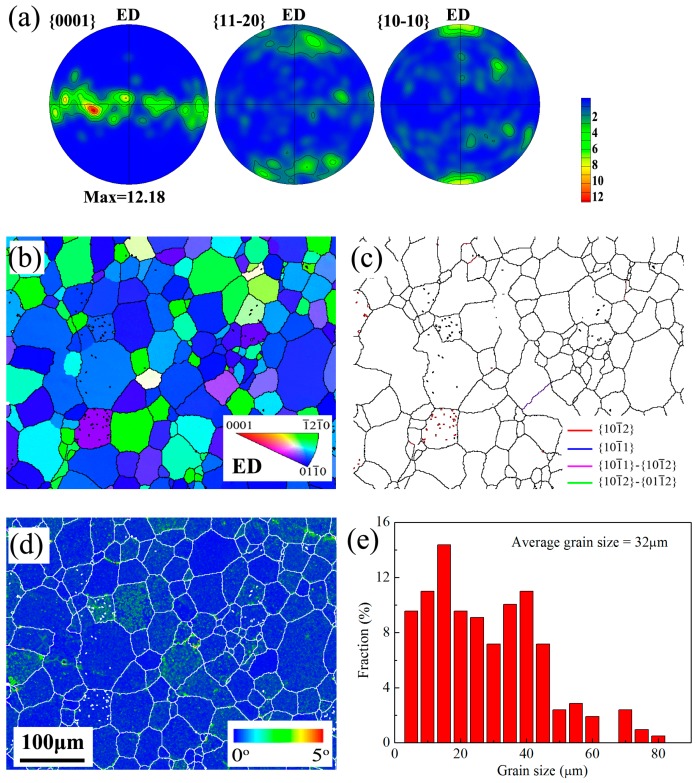
Microstructural characteristics of the as-solution AZ91 rod: (**a**) pole figures; (**b**) IPF map; (**c**) GB and TB map; (**d**) KAM map and (**e**) grain size distribution. The average grain size was calculated from the number fraction.

Figure 2 shows the EBSD maps and pole figures of SS samples. EBSD maps are shown as inverse pole figure (IPF) maps, grain boundary and twin boundary (GB and TB) maps and kernel average misorientation (KAM) maps. The grain size distribution can also be obtained by EBSD data [31] and shown in Figure 2e. The SS sample has a uniform microstructure with an average grain size of approx. 32 μm and a typical extrusion <10-10> fiber texture (i.e., <10-10> // ED texture). GB and TB maps indicate the SS sample is free of twin structures.

**Figure 3:**
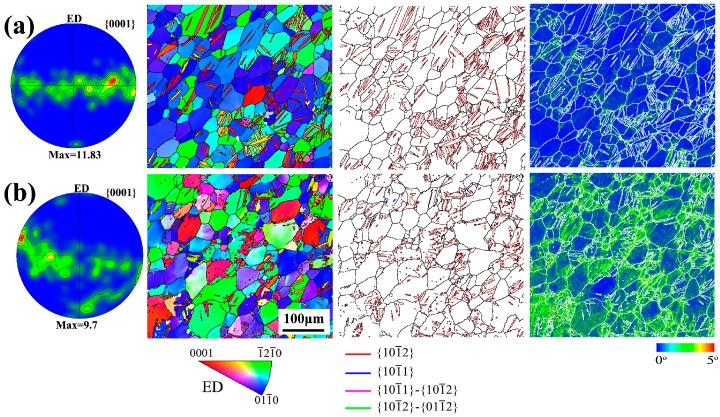
Pole figures and EBSD maps of the torsion-deformedAZ91 rod: (**a**) center position; (**b**) edge position.

**Figure 4:**
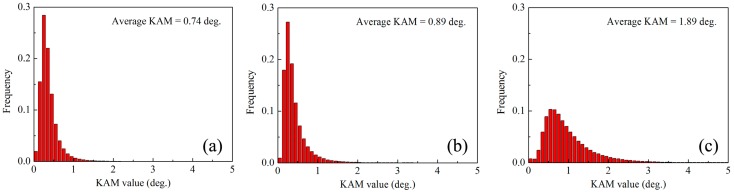
KAM distribution of various samples: (**a**) SS; (**b**) center position of ST and (**c**) edge position of ST.

It has been reported that torsion deformation can introduce a gradient strain along the radius direction [22]. Thus, to clearly investigate the microstructure evolution during torsion, the edge and center positions on cross-section of rods were characterized by EBSD for ST sample, as shown in Figure 3. After torsion, both edge and center positions contain a large number of {10-12} extension twins. The area fraction can be calculated by using EBSD data. It is found that the area fraction of twins in edge position (~22%) is far higher than that in center position (~8%) for ST sample. The Kernel Average Misorientation (KAM) was also calculated from the EBSD analysis. KAM cartography represents the mean angle between the crystallographic orientation of each pixel and those of its eight nearest neighbors (misorientations below 5° are included in the calculation of KAM). KAM maps are used to analyze the distribution and amount of dislocations, which quantifies the local lattice curvature [32]. Figure 4 shows the KAM distribution of various samples. For SS sample, the KAM analysis shows a very low misorientation exists in each grain. Torsion deformation increases the KAM. It shows that the KAM in edge position is remarkably higher than that in center position for the ST sample. It also indicates that torsion deformation generates more dislocations in the edge position than in the center position.

**Figure 5:**
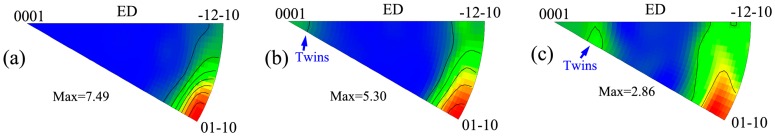
Inverse pole figures of (**a**) SS; (**b**) center position of ST and (**c**) edge position of ST.

Figure 3 also indicates that torsion deformation can cause textural change. To better investigate the effect of torsion deformation on the orientation relationship between *c*-axis and ED, the inverse pole figures of various samples are shown in Figure 5. Extruded AZ91 rod contains extrusion fiber texture (i.e., <10-10> // ED texture), so the *c*-axes of most grains are perpendicular to the ED, as shown in Figure 5a. Both {10-12} twinning and slips could arouse the textural change. For the ST sample, the texture intensity of twin orientation is very weak owing to low twin fraction. For un-twinned regions, slip deformation can also cause the textural change, as shown in Figure 5b,c. For the center position with low strain, un-twinned region retains the extrusion texture. For the edge position with maximum shear strain, the basal pole has a more widespread distribution and tends to rotate towards ED. Basal pole peak of un-twinned grains at the edge position locates at the ~80° from ED.

Clearly, torsion deformation can generate gradient microstructure on the cross-section of as-extruded Mg rod. From center to edge on the cross-section of rod, both stored dislocations and {10-12} twins gradually increase and the basal pole of texture tends to rotate towards ED direction.

### 3.2. Precipitation Behavior

**Figure 6:**
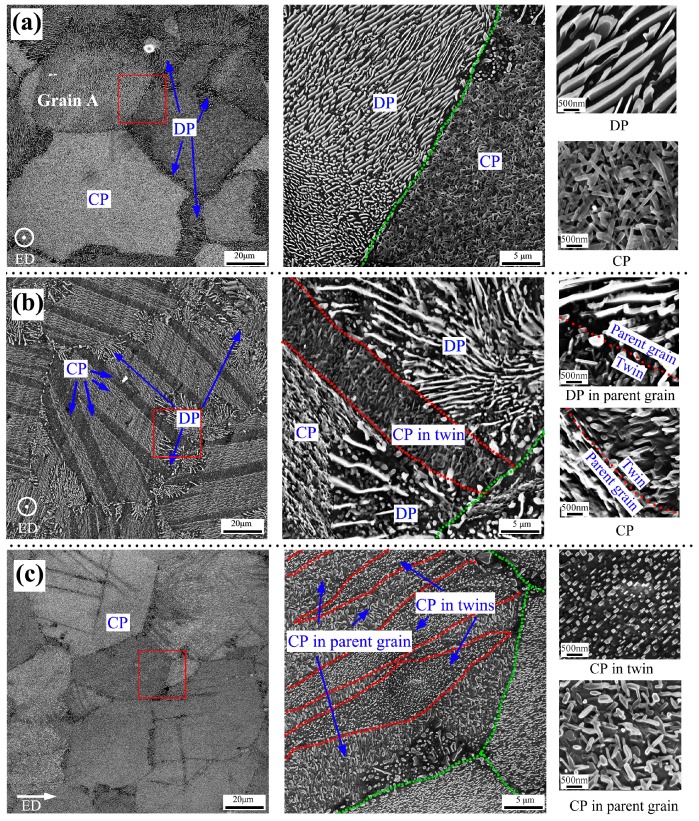
SEM images of (**a**) SA; (**b**) center position of STA and (**c**) edge position of STA. Green dotted lines and red dotted lines outline the grain boundaries and twin boundaries, respectively. The images at the middle are taken at higher magnification from the corresponding marked areas, and those at the right side show the detail observation of two types of precipitates, i.e., CP and DP, at high magnification.

Figure 6 shows SEM images of various aged samples. It is observed that Mg_17_Al_12_ phases exhibit a non-uniform distribution in SA sample. The coarse discontinuous precipitates (DP) and fine continuous precipitates (CP) usually simultaneously precipitate within the same grain, as shown in Figure 6a. Discontinuous precipitates with lamellar-shape usually mainly concentrate nearby grain boundaries and even could almost occupy the whole grain (e.g., Grain A in Figure 6a). Through the statistics of five SEM images with an area of about 17689 μm^2^, the area fractions of DP (*f*_-DP_) and CP (*f*_-CP_) were evaluated and listed in Table 2. It is found that *f*_-DP_ is 48% for the SA sample. Torsion deformation can generate a gradient microstructure on the cross-section of AZ91 rod, as shown in Figure 3. Thus, the SEM was carried out on the center and the edge positions to investigate the precipitation behavior during aging. For the center position, it is shown that the precipitates in the un-twinned regions of the STA sample exhibits a similar precipitation feature with SA sample. However, aging treatment can induce a uniform continuous precipitation within twins and twin structures are the favorable nucleation sites only for the continuous precipitation, as shown in Figure 6b. Thus, the *f*_-DP_ is reduced to 34% in center position of the STA sample. For the edge position of the STA sample, both un-twinned and twinned regions can be the favorable nucleation sites for the continuous precipitation (Figure 6c), resulting in that the coarse lamellar-shape discontinuous precipitates are strongly inhibited (*f*_-DP_ = 11%). Moreover, it is noticed that the continuous precipitates in un-twinned and twinned regions of each grain exhibit different morphologies. This can be attributed to the different observation crystallographic plane between un-twinned and twinned regions due to that {10-12} twining causes a grain rotation of ~86.3° [33]. Clearly, torsion deformation can promote the continuous precipitates and generate a gradient precipitation feature.

### 3.3. Tensile and Compressive Properties

**Figure 7:**
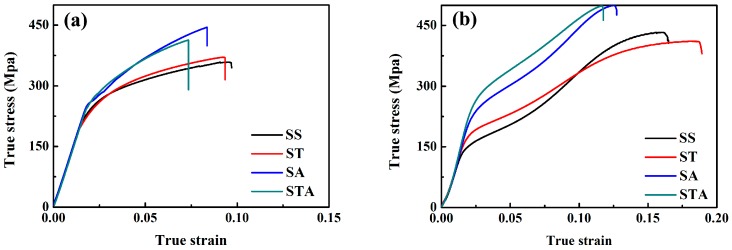
True stress and true strain curves of various samples: (**a**) tension and (**b**) compression.

Figure 7 exhibits the tensile and compressive curves along the ED of AZ91 rods that underwent various treatments. Table 3 lists the detail mechanical properties. For SS sample, compressive yield strength (146 MPa) is far lower than tensile yield strength (231 MPa). Clearly, the SS sample exhibits a large yield asymmetry (CYS/TYS = 0.63). Torsion deformation can increase compressive yield strength (by 29 MPa) while decreasing tensile yield strength (by 11 MPa), resulting in the improvement of yield asymmetry (CYS/TYS = 0.80). Aging treatment can generate strong precipitation hardening effect on present AZ91 alloys. Yield strengths of SS sample are increased by 33 MPa and 80 MPa by direct aging for tension and compression, respectively. Table 3 also shows that the subsequent aging treatment after torsion deformation can generate higher hardening effect on the SS sample compared with direct aging. The increments in yield strength via combined torsion deformation and aging treatment are 32 MPa and 115 MPa, respectively, for tension and compression. Moreover, aging treatment can also reduce the yield asymmetry and combining torsion deformation and aging treatment almost eliminates the yield asymmetry. The CYS/TYS are 0.86 and 0.99, respectively, for SA and STA samples. It can therefore be concluded that the combined use of torsion deformation and aging treatment can significantly enhance the yield strength and eliminate the yield asymmetry of the present extruded AZ91 rod.

## 4. Discussion

In this study, torsion deformation can introduce a gradient distribution of microstructure. It could be attributed to the nature of torsion deformation, i.e., inhomogeneous deformation. Torsion can generate a gradient strain within the samples. The equivalent strain in torsion can be calculated as [34]:

γ = 2π*Nr*/*l* (1)

(2) $$\varepsilon =\gamma /\sqrt{3}$$

where γ is shear strain, *N* is the number of rotation, *r* is the radial position in the sample, *l* is the sample length and ε is the equivalent strain. This shows that torsion deformation can generate a gradient distribution of strain and the equivalent strain linearly increases from core to surface of rod. As discussed in Section 3.1, with increasing shear strain, both dislocation density and twin amount increase and the *c*-axis of texture tends to rotate towards ED direction. Previous reports have revealed that dislocation slips are dominant deformation mechanism of torsion deformation for extruded Mg alloys [23,30,35]. Thus, the high-density of dislocations exist in un-twinned regions of edge position for ST sample, as shown in Figure 3b. Slip deformation via torsion can also induce the *c*-axis of texture rotate toward ED during torsion. The ideal orientation for Mg alloys under torsional shear deformation is known as B fiber texture with *c*-axis// ED [36]. For extruded Mg alloy rods, the *c*-axes of most grains are perpendicular to ED. On this occasion, torsion deformation will induce the *c*-axes to rotate towards ED [37].

For the SS sample, the coarse discontinuous precipitates and fine continuous precipitates can simultaneously precipitate, as shown in Figure 6a. It is known that the continuous and discontinuous precipitations compete in an intricate manner because they nucleate and grow at different rates and with different mechanisms [38]. Figure 6 shows that the precipitation behavior of AZ91 alloys with high content of Al element can be influenced by cold torsion deformation. For the ST sample, both extension twins and dislocations have been introduced during torsion deformation. It has been reported that uniform continuous Mg_17_Al_12_ phases are favorably formed within extension twins introduced by pre-strain [8,11,13,17]. For the ST sample, both center and edge positions contain extension twins. After aging, twinned regions only contain dense continuous precipitates. The result is consistent with previous reports [8,11,13,17]. It is known that center and edge positions suffer the lowest and highest torsion strain, respectively. Thus, it can be inferred that continuous precipitation in twins is independent from the pre-strain amount and twin size. Once the twins are introduced by pre-strain, the uniform continuous precipitation can be formed in twins during subsequent aging. However, for un-twinned regions, center and edge positions exhibit distinct precipitation behavior, as shown in Figure 6b,c. For center position, the un-twinned regions have a similar precipitation behavior with SA sample. However, at edge position of ST, uniform continuous Mg_17_Al_12_ phases can also be favorably formed within un-twinned regions as well as within extension twins and the coarse discontinuous precipitates can be almost completely inhibited.

**Figure 8:**
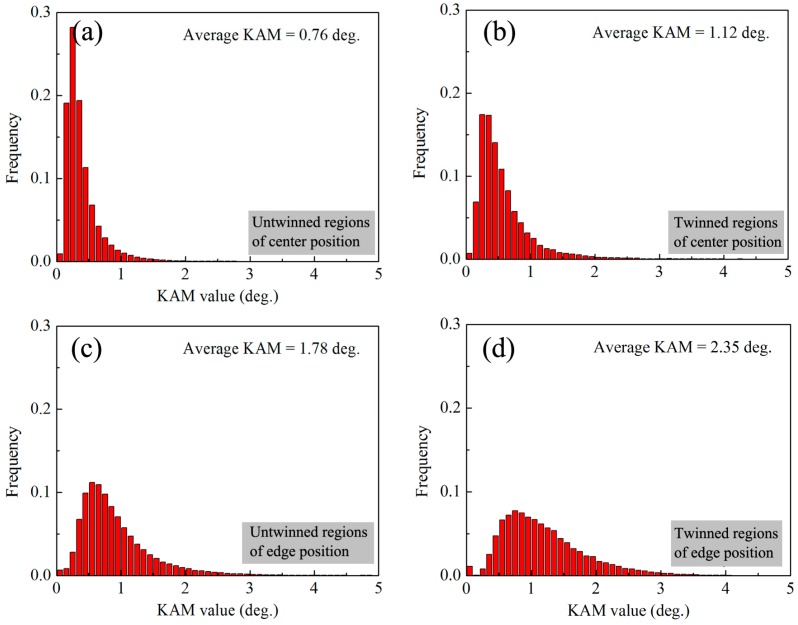
KAM value distribution of ST sample: (**a**) un-twinned regions of center position; (**b**) twinned regions of center position; (**c**) un-twinned regions of edge position and (**d**) twinned regions of edge position.

Generally, lattice defects via pre-deformation can promote the nucleation and suppress coarsening of precipitates [8,9,10,11,13,17]. Thus, precipitation characteristics could be related with the distribution of lattice defects. Figure 8 shows the KAM distribution of various regions in ST sample. It shows that average KAM in twins is still higher than that in un-twinned region for center and edge positions. Some studies have found that the lattice defects including dislocations and stacking faults in twins could be closely associated with migrations of twin boundaries [8,39,40,41]. Thus, twinning deformation could introduce a large number of lattice defects inside twins, which can provide more heterogeneous nucleation sites for precipitation. By contrast, lattice defects (i.e., dislocations) of un-twinned regions results from dislocation slips and are more dependent on the plastic strain. Moreover, dislocation slips could also occur in pre-existed twins during deformation. Therefore, with increasing strain, both the lattice defects of twinned and un-twinned regions could increase. As shown in Figure 8, from center to edge on the cross-section of ST sample, average KAM increases from 0.76° to 1.78° for un-twinned regions and 1.12° to 2.35° for twinned regions. Previous research has pointed out dislocations can facilitate nucleation of precipitates and enhance the number density of precipitates during subsequent ageing treatment [9]. Thus, it is considered that the high density of dislocations in un-twinned regions of edge position can also promote continuous precipitation and inhibit coarse discontinuous precipitation. For the center position, torsion deformation only slightly increases the average KAM of un-twinned regions (from 0.74 to 0.76) owing to low strain, as shown in Figure 4a and Figure 8a. Thus, un-twinned regions of center position in ST sample exhibit a similar precipitation behavior with SS sample. Clearly, precipitation behavior of AZ91 rod is closely dependent on the amount of twins and dislocations. As shown in Figure 3, torsion deformation generates a gradient distribution of twins and dislocations. Therefore, aging after torsion deformation can generate a gradient distribution of precipitate characteristics.

Figure 7 shows both torsion deformation and aging treatment can greatly influence the mechanical properties. To understand the effect of the change in microstructure via torsion and aging on the tensile and compressive yield strength, a linear superposition rule is assumed to explain the contributions of various factors to the yield strength [15]:

σ_0.2_ = σ_0_ + σ_HP_ + σ_d_ + σ_ppt_ + σ_ori_

where σ_0_ is a frictional contribution, σ_HP_ is the contribution from refinement hardening, σ_d_ is the contribution from dislocation hardening, σ_ppt_ is the contribution from precipitation hardening and σ_ori_ is the contribution from orientation hardening.

Torsion deformation can introduce dislocations and twin lamellae and cause textural change. Dislocations and twin boundaries can generate dislocation hardening and refinement hardening effects, respectively [21]. Thus, it could be expected that torsion deformation can enhance the tensile and compressive yield strengths. In fact, present torsion deformation increases compressive yield strength, while reduces tensile yield strength, as shown in Figure 7. The possible reason is textural change during torsion [22].

Textural change will influence the Schmid factor (SF) of dominated deformation modes. For extruded magnesium alloy rods with fiber texture (i.e., *c*-axis⊥ED), the basal slip with the lowest critical resolved shear stress (CRSS) is hard to activate owing to the low SF for deformation along ED. Under this occasion, it is generally considered that yielding in tension and compression along ED are dominated by prismatic slip and extension twinning, respectively. The large yield asymmetry of as-extruded magnesium alloy is attributed to the fact that the CRSS for extension twinning is far less than that for prismatic slip [4]. Torsion deformation causes the c-axis of the texture to rotate towards ED and the largest angle of rotation can achieve ~10° in the edge position. The textural change via torsion will increase SF of basal slip during deformation along ED, resulting in the reduction of tensile yield strength. By contrast, it could exhibit a smaller influence on the compressive yield stress due to that the difference of CRSS between basal slip and {10-12} twinning is lower than that between basal slip and prismatic slip [22,42,43]. Thus, textural change via torsion can effectively reduce yield asymmetry of Mg alloy rods.

**Figure 9:**
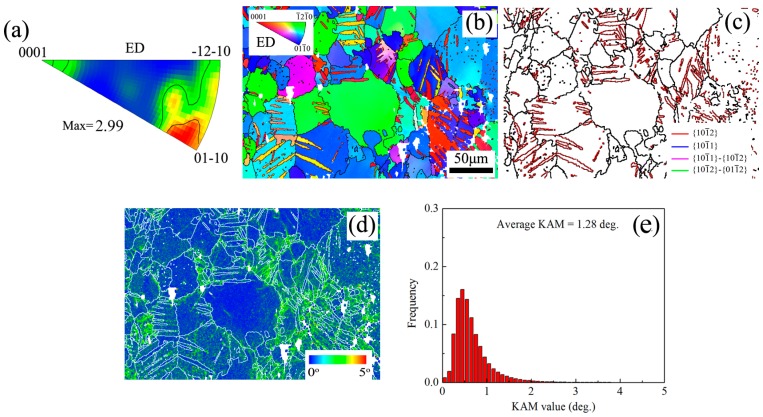
Microstructure characteristics in edge position of STA sample: (**a**) inverse pole figure (**b**) IPF map; (**c**) GB + TB map; (**d**) KAM map and (**e**) KAM distribution figure.

Figure 7 shows that aging treatment can enhance the tensile and compressive yield strength. Moreover, it can also be found that increments of yield strength via aging treatment are 33 MPa and 80 MPa for tension and compression of SS sample, respectively, and are 39 MPa and 86 MPa for tension and compression of ST sample, respectively. It shows that ST sample has higher aging hardening effect compared with SS sample. In fact, for ST sample, the increment of yield strength via aging precipitation may be underestimated due to the loss of dislocations from the matrix during aging [15,21]. Figure 9 shows the EBSD map of edge position in STA sample. It indicates that aging treatment at 180 °C does not arouse static recrystallization, but reduces the average KAM from 1.89° to 1.28°. Thus, aging treatment can retain twin structure and texture, while reduce the dislocation hardening effect. It can be inferred that torsion deformation enhances aging hardening effect. As shown in Figure 6, after torsion, the fine and high-density of continuous precipitates can be readily precipitated within the twinned regions of lower strain layer and the twinned and un-twinned regions of high strain layer. Thus, torsion deformation promotes fine continuous precipitates and suppresses coarse continuous precipitates. Promotion of continuous precipitation reduces interparticle spacing and increases number density of precipitates, resulting in the enhancement of the aging hardening effect [8].

Both torsion deformation and aging treatment can reduce the yield asymmetry. Firstly, textural change via torsion deformation can reduce the yield asymmetry [22]. Moreover, twin lamellae can subdivide grains and lead to refinement of grains [21]. Barnett et al. [44] reported that the refinement grain can harden the twinning-dominated yield stress more than slip-dominated yield stress. Thus, the textural change and generation of twin lamellae can both be responsible for the improvement of yield asymmetry of ST sample [21,22]. Aging treatment can precipitate the Mg_17_Al_12_ phase with basal plates in Mg-Al alloys. It has been reported that the Mg_17_Al_12_ phase can generate a higher Orowan hardening effect on extension twinning than on prismatic slip, leading to a reduction in yield asymmetry [4]. The subsequent aging treatment after torsion not only precipitates higher-density continuous Mg_17_Al_12_ precipitates, but also retains the torsion-deformed texture and twin lamellae. It could be the reason why the yield asymmetry of STA is almost eliminated.

In this study, it has proved that torsion deformation is an effective method to improve the mechanical properties of extruded AZ91 rods. It shows that both twins and dislocations via torsion deformation can be favorable nucleation sites for the continuous precipitations. It can contribute to the enhancement of aging-hardening response and the reduction of yield asymmetry of extruded AZ91 rod. Recently, some works have focused on the enhancement of age-hardening response of Mg alloys by pre-inducing dislocations or twins [8,9,10,11,12,13]. Some simple plastic deformation techniques (e.g., pre-tension, pre-compression and pre-rolling) have been employed to achieve this purpose [8,9,10,11,12,13]. Moreover, severe plastic deformation processes (e.g., high-pressure torsion, equal-channel angular pressing and accumulative roll bonding) can impose a large strain to introduce a high density of lattice defects in materials and are potential methods to enhance the aging hardening response [45]. In contrast, free-end torsion deformation belongs to a simple plastic deformation technique and has some superiority to tailor the microstructure of rod-shaped materials. For example, free-end torsion has little influence on the shape and size of rods and has no limitation of sample size [30,37]. Moreover, it can also achieve a large plastic strain without rupture or strain localization and introduce gradient microstructure [22,23,24,25,26,27,28]. Recent reports show preparation of gradient microstructure can provide a new sight for superior ductility-strength combination and the improvement of surface hardness, corrosion property and fatigue property [27,46,47]. Therefore, as a simple plastic deformation technique, free-end torsion deformation is considered to be an effective and low-cost method to fabricate the high-property rod-shaped structure parts (e.g., bearing and rotor).

## 5. Conclusions

In this study, an AZ91 magnesium alloy rod was used to investigate the effects of torsion deformation on microstructure and subsequent aging behavior. Moreover, tension and compression properties of various samples were also discussed. Following conclusions can be drawn:

1. The SS sample has a uniform microstructure and typical fiber texture. Torsion deformation can generate gradient microstructure evolution on the cross-section of as-extruded AZ91 rod. After torsion, from the center to the edge on the cross-section of rod, both stored dislocations and area fraction of {10-12} twins gradually increase, and the basal pole of the texture tends to rotate towards ED direction.
2. Direct aging usually generates coarse discontinuous precipitates and fine continuous precipitates simultaneously. Torsion deformation can promote continuous precipitation. Both twin structures and dislocations via torsion deformation can be effective microstructures for the nucleation of continuous precipitates.
3. Twin structures are favorable nucleation sites only for the continuous precipitation, while the precipitation behavior of un-twinned regions largely depends on the strain amount. High strain can generate a mass of dislocations to promote continuous precipitation in un-twinned regions. Thus, ST sample exhibits gradient precipitation characteristics due to the gradient distribution of twins and dislocations.
4. SS sample exhibits a large tension-compression yield asymmetry. Aging treatment can enhance the tensile and compressive yield strength simultaneously and reduce yield asymmetry. Torsion deformation can also reduce the yield asymmetry owing to the generation of twin lamellae and textural change. Moreover, torsion deformation can enhance the aging hardening effect. Finally, combined use of torsion deformation and aging treatment can effectively enhance the yield strength and almost eliminate the yield asymmetry of the present extruded AZ91 rod.

## Figures and Tables

**Table 1:** Extruded AZ91 rods subjected to various processing histories.

Samples	Processing History
SS	As-solution extruded rod
SA	Direct aging
ST	Torsion deformation
STA	Torsion deformation and then aging

**Table 2:** Average area fractions of CP and DP in various samples.

Samples	SA	STA-Center	STA-Edge
*f*_-DP_	0.48	0.34	0.11
*f*_-CP_	0.52	0.66	0.89

**Table 3:** Yield strength (YS), peak strength (PS), uniform elongation (UE) and CYS/TYS of various samples.

Samples	Loading Conditions	YS (MPa)	PS (MPa)	UE (%)	CYS/TYS
SS	Ten.	231	358	7.9	0.63
	Comp.	146	433	14.1	
ST	Ten.	220	372	7.6	0.80
	Comp.	175	411	16.6	
SA	Ten.	264	446	6.4	0.86
	Comp.	226	502	10.6	
STA	Ten.	263	427	6.0	0.99
	Comp.	261	500	9.7	

## References

[1] Wang F., Bhattacharyya J.J., Agnew S.R. (2016). Effect of precipitate shape and orientation on Orowan strengthening of non-basal slip modes in hexagonal crystals, application to magnesium alloys. Mater. Sci. Eng. A.

[2] Nie J.-F. (2012). Precipitation and Hardening in Magnesium Alloys. Metall. Mater. Trans. A.

[3] Stanford N., Geng J., Chun Y.B., Davies C.H.J., Nie J.F., Barnett M.R. (2012). Effect of plate-shaped particle distributions on the deformation behaviour of magnesium alloy AZ91 in tension and compression. Acta Mater..

[4] Robson J.D., Stanford N., Barnett M.R. (2011). Effect of precipitate shape on slip and twinning in magnesium alloys. Acta Mater..

[5] Nie J.F. (2003). Effects of precipitate shape and orientation on dispersion strengthening in magnesium alloys. Scr. Mater..

[6] Xin R., Song B., Zeng K., Huang G., Liu Q. (2012). Effect of aging precipitation on mechanical anisotropy of an extruded Mg-Y-Nd alloy. Mater. Des..

[7] Lv C., Liu T., Liu D., Jiang S., Zeng W. (2012). Effect of heat treatment on tension-compression yield asymmetry of AZ80 magnesium alloy. Mater. Des..

[8] Wang C., Xin R., Li D., Song B., Wu M., Liu Q. (2017). Enhancing the age-hardening response of rolled AZ80 alloy by pre-twinning deformation. Mater. Sci. Eng. A.

[9] Li R., Xin R., Chapuis A., Liu Q., Fu G., Zong L., Yu Y., Guo B., Guo S. (2016). Effect of cold rolling on microstructure and mechanical property of extruded Mg-4Sm alloy during aging. Mater. Charact..

[10] Zheng K.Y., Dong J., Zeng X.Q., Ding W.J. (2008). Effect of pre-deformation on aging characteristics and mechanical properties of a Mg-Gd-Nd-Zr alloy. Mater. Sci. Eng. A.

[11] Yang P., Wang L.-N., Xie Q.-G., Li J.-Z., Ding H., Lu L.-L. (2011). Influence of deformation on precipitation in AZ80 magnesium alloy. Int. J. Miner. Metall. Mater..

[12] Čížek J., Procházka I., Smola B., Stulíková I., Očenášek V. (2007). Influence of deformation on precipitation process in Mg-15wt.%Gd alloy. J. Alloys Compd..

[13] Ye J., Lin X.-P., Zhao T.-B., Liu N.-N., Xie H.-B., Niu Y., Teng F. (2016). Influence of pre-strain on the aging hardening effect of the Mg-9.02Zn-1.68Y alloy. Mater. Sci. Eng. A.

[14] Hong S.-G., Park S.H., Lee C.S. (2010). Role of {10-12} twinning characteristics in the deformation behavior of a polycrystalline magnesium alloy. Acta Mater..

[15] Song B., Xin R., Sun L., Chen G., Liu Q. (2013). Enhancing the strength of rolled ZK60 alloys via the combined use of twinning deformation and aging treatment. Mater. Sci. Eng. A.

[16] Chen H., Liu T., Zhang Y., Song B., Hou D., Pan F. (2016). The yield asymmetry and precipitation behavior of pre-twinned ZK60 alloy. Mater. Sci. Eng. A.

[17] Zhang Y., Liu T., Ding X., Xu S., He J., Chen H., Pan F., Lu L. (2014). The precipitation behavior of a pretwinned Mg-6Al-1Zn alloy and the effect on subsequent deformation. J. Mater. Res..

[18] Song B., Guo N., Liu T., Yang Q. (2014). Improvement of formability and mechanical properties of magnesium alloys via pre-twinning: A review. Mater. Des..

[19] Song B., Xin R., Zheng X., Chen G., Liu Q. (2015). Activation of multiple twins by pre-tension and compression to enhance the strength of Mg-3Al-1Zn alloy plates. Mater. Sci. Eng. A.

[20] Park S.H., Hong S.-G., Lee J.-H., Kim S.-H., Cho Y.-R., Yoon J., Lee C.S. (2017). Effects of pre-tension on fatigue behavior of rolled magnesium alloy. Mater. Sci. Eng. A.

[21] Song B., Xin R., Chen G., Zhang X., Liu Q. (2012). Improving tensile and compressive properties of magnesium alloy plates by pre-cold rolling. Scr. Mater..

[22] Guo N., Song B., Guo C., Xin R., Liu Q. (2015). Improving tensile and compressive properties of magnesium alloy rods via a simple pre-torsion deformation. Mater. Des..

[23] Song B., Zhao H., Chai L., Guo N., Pan H., Chen H., Xin R. (2016). Preparation and characterization of Mg alloy rods with gradient microstructure by torsion deformation. Met. Mater. Int..

[24] Song B., Guo N., Xin R., Pan H., Guo C. (2016). Strengthening and toughening of extruded magnesium alloy rods by combining pre-torsion deformation with subsequent annealing. Mater. Sci. Eng. A.

[25] Wang J., Zhang D., Li Y., Xiao Z., Fouse J., Yang X. (2015). Effect of initial orientation on the microstructure and mechanical properties of textured AZ31 Mg alloy during torsion and annealing. Mater. Des..

[26] Chen C., Beygelzimer Y., Toth L.S., Estrin Y., Kulagin R. (2016). Tensile Yield Strength of a Material Preprocessed by Simple Shear. J. Eng. Mater. Technol..

[27] Wei Y., Li Y., Zhu L., Liu Y., Lei X., Wang G., Wu Y., Mi Z., Liu J., Wang H., Gao H. (2014). Evading the strength-ductility trade-off dilemma in steel through gradient hierarchical nanotwins. Nat. Commun..

[28] Guo N., Song B., Yu H., Xin R., Wang B., Liu T. (2016). Enhancing tensile strength of Cu by introducing gradient microstructures via a simple torsion deformation. Mater. Des..

[29] Jung I.C., Kim Y.K., Cho T.H., Oh S.H., Kim T.E., Shon S.W., Kim W.T., Kim D.H. (2013). Suppression of discontinuous precipitation in AZ91 by addition of Sn. Met. Mater. Int..

[30] Guo X.Q., Wu W., Wu P.D., Qiao H., An K., Liaw P.K. (2013). On the Swift effect and twinning in a rolled magnesium alloy under free-end torsion. Scr. Mater..

[31] Toth L.S., Biswas S., Gu C., Beausir B. (2013). Notes on representing grain size distributions obtained by electron backscatter diffraction. Mater. Charact..

[32] Britton T.B., Birosca S., Preuss M., Wilkinson A.J. (2010). Electron backscatter diffraction study of dislocation content of a macrozone in hot-rolled Ti-6Al-4V alloy. Scr. Mater..

[33] Celotto S. (2000). Tem study of continuous precipitation in Mg-9 Wt%Al-1 Wt%Zn alloy. Acta Mater..

[34] Guo N., Luan B., Liu Q. (2013). Influence of pre-torsion deformation on microstructures and properties of cold drawing pearlitic steel wires. Mater. Des..

[35] Kabirian F., Khan A.S., Gnäupel-Herlod T. (2015). Visco-plastic modeling of mechanical responses and texture evolution in extruded AZ31 magnesium alloy for various loading conditions. Int. J. Plast..

[36] Beausir B., Tóth L.S., Neale K.W. (2007). Ideal orientations and persistence characteristics of hexagonal close packed crystals in simple shear. Acta Mater..

[37] Biswas S., Beausir B., Toth L.S., Suwas S. (2013). Evolution of texture and microstructure during hot torsion of a magnesium alloy. Acta Mater..

[38] Braszczyńska-Malik K.N. (2009). Discontinuous and continuous precipitation in magnesium-aluminium type alloys. J. Alloys Compd..

[39] Pan H., Huang Q., Qin G., Fu H., Xu M., Ren Y., She J., Song B., Li B. (2017). Activations of stacking faults in the calcium-containing magnesium alloys under compression. J. Alloys Compd..

[40] Zhang D., Zheng B., Zhou Y., Mahajan S., Lavernia E.J. (2014). Prism stacking faults observed contiguous to a {10-12} twin in a Mg-Y alloy. Scr. Mater..

[41] Sun Q., Zhang X., Shu Y., Tan L., Liu Q. (2016). Two types of basal stacking faults within {10-12} twin in deformed magnesium alloy. Mater. Lett..

[42] Park S.H., Hong S.-G., Lee C.S. (2013). In-plane anisotropic deformation behavior of rolled Mg-3Al-1Zn alloy by initial {10-12} twins. Mater. Sci. Eng. A.

[43] Yin D.L., Wang J.T., Liu J.Q., Zhao X. (2009). On tension-compression yield asymmetry in an extruded Mg-3Al-1Zn alloy. J. Alloys Compds..

[44] Barnett M.R., Keshavarz Z., Beer A.G., Atwell D. (2004). Influence of grain size on the compressive deformation of wrought Mg-3Al-1Zn. Acta Mater..

[45] Estrin Y., Vinogradov A. (2013). Extreme grain refinement by severe plastic deformation: A wealth of challenging science. Acta Mater..

[46] Lu K. (2014). Making strong nanomaterials ductile with gradients. Science.

[47] Lu K. (2015). Gradient nanostructured materials. Acta Metall. Sin..

